# Fabrication of Radial Array Transducers Using 1-3 Composite via a Bending and Superposition Technique

**DOI:** 10.3390/mi15111363

**Published:** 2024-11-11

**Authors:** Chong Li, Jing Zhu, Ruimin Chen

**Affiliations:** 1 School of Intelligent Manufacturing and Control Engineering, Shanghai Polytechnic University, Shanghai 201209, China; 2 Research Center for Novel Computing Sensing and Intelligent Processing, Zhejiang Lab, Hangzhou 311100, China; zhujing@zhejianglab.org (J.Z.); ruimin.chen@zhejianglab.org (R.C.)

**Keywords:** 1-3 composite, radial array transducer, flexible circuit board, bending-and-superposition

## Abstract

Piezoelectric composite materials, combining the advantages of both piezoelectric materials and polymers, have been extensively used in ultrasonic transducers. However, the pitch size of radial array ultrasonic transducers normally exceeds one wavelength, which limits their performance. In order to minimize grating lobes of current radial transducers and then increase their imaging resolution, a 2.5 MHz 1-3 composite radial array transducer with 64 elements and 600 μm pitch was designed and fabricated by combining flexible circuit board and using a bending-and-superposition method. All the array elements were connected and actuated via the customized circuit board which is thin and soft. The kerf size is set to be 1/3 wavelength. PZT-5H/epoxy 1-3 composite was used as an active material because it exhibits an ultrahigh electromechanical coupling coefficient (*k_t_* = 0.74), a very low mechanical quality factor (*Q_m_* = 11), and relatively low acoustic impedance (*Z_c_* = 13.43 MRayls). The developed radial array transducer exhibited a center frequency of 2.72 MHz, an average −6 dB bandwidth of 36%, an insertion loss of 31.86 dB, and a crosstalk of −26.56 dB between the adjacent elements near the center frequency. These results indicate that the bending-and-superposition method is an effective way to fabricate radial array transducers by binding flexible circuit boards. Furthermore, the utilization of tailored flexible circuitry boards presents an effective approach for realizing reductions in crosstalk level (CTL).

## 1. Introduction

Capsule endoscopy has emerged as a prominent non-invasive diagnostic method for assessing the health of the gastrointestinal (GI) tract. It utilizes a miniaturized capsule-shaped device equipped with a camera system to capture images of the GI tract. The captured images are subsequently transmitted to external recording devices for further analysis by a physician [1]. However, the observation is limited to superficial information on the GI tract. The remarkable ability of endoscopic ultrasound (EUS) to image deep tissues has sparked considerable interest in its integration with capsules, aiming to augment diagnostic depth. Traditional EUS systems feature a single-element ultrasonic transducer at the catheter’s tip. Single-element transducers, while simpler in construction compared to array transducers, are constrained by a fixed focal point. As a result, they are capable of targeting only one area at a time, which requires frequent mechanical realignment to achieve comprehensive 360-degree scanning and subsequently construct two-dimensional images. It also means that conventional single-element EUS systems should incorporate an electromagnetic motor that drives a flexible shaft, allowing the transducer to mechanically rotate and acquire a complete 360-degree perspective. However, electromagnetic motors, typically large and complex, are often situated far from the transducer, thereby limiting the rotational velocity of EUS systems. Then, the imaging frame rate is also constrained by the time-consuming nature of mechanical scanning techniques [2,3,4]. Furthermore, integrating the single-element imaging system directly into the capsule is challenged by the limited space available within it.

To solve these problems, radial array ultrasound transducers have attracted increasing attention. The foundational principle of achieving focus with radial array transducers relies on the application of exact time delays to the excitation signals of each constituent element within the array. This method facilitates the coherent summation of acoustic waves at a designated focal point, culminating in the formation of an intensified acoustic field. This also suggests that radial array transducers are capable of enabling 360-degree electronic scanning, eliminating the necessity for supplementary mechanical components [3,4,5,6,7]. Hence, radial array transducers hold enormous potential for quicker image acquisition and enhanced clarity in capsule endoscopy. Nevertheless, the successful implementation of these transducers still faces several challenges that need to be addressed urgently. Firstly, to achieve the necessary resolution and image quality, a sufficient number of piezoelectric elements must be accommodated within the confined space of the capsule, necessitating a reduction in the size of individual transducer elements to specific dimensions. Secondly, piezoelectric ceramics, which serve as the core excitation components for ultrasound transducers owing to their superior electromechanical properties, are inherently brittle. This brittleness poses a significant risk of element fracture during the rolling process needed to form the cylindrical array. Consequently, the complexity of fabricating radial array transducers hinders their broader application.

Although piezopolymers like polyvinylidene fluoride (PVDF) exhibit favorable acoustic impedance matching with human tissues and remarkable flexibility, their utilization as the active material in radial array transducers is constrained by their inherently low piezoelectric coefficients [2]. To enhance the performance of piezoelectric materials for their suitability in radial array transducers, composites are developed. Among them, 1-3 composite, consisting of piezoelectric rods and filled passive epoxy resin, are a more suitable option for the active material of radial ultrasonic transducers because of their superior electromechanical coupling coefficient and enhanced flexibility. Additionally, the acoustic impedance of composites closely matches that of human tissues, facilitating efficient energy transfer and minimizing impedance mismatch-induced losses. Over the past few decades, intensive research efforts have been devoted to the development of radial array transducers employing 1-3 composite materials. Zhou et al. reported a radial array transducer that uses PMN-PT single crystal/epoxy 1-3 composite and is fabricated by the wrapping method [2]. Zhang et al. fabricated a 6.8-MHz 128-element endoscopic ultrasonic transducer based on high-performance PMN-PT 1-3 composite [6]. Subsequently, Zhang et al. explored and constructed an 84 × 5 element 1.5-dimensional circular array transducer [8]. Nevertheless, the element pitch in these transducers exceeds a wavelength in the medium, which is relatively large and may lead to the formation of grating lobes at the operation frequency. Then, the performance of the imaging system is degraded by introducing errors and artifacts in the final image. Moreover, the crosstalk phenomenon between adjacent elements existing in the above-developed radial array transducers has not been thoroughly investigated.

This study is dedicated to developing a miniaturized radial array transducer made of 1-3 piezoelectric composite for integration into an endoscopic capsule. In light of the dimension features of existing endoscopy capsules, a 2.5 MHz, 64-element radial ultrasonic transducer was developed using a bending-and-superposition method. This transducer, crafted from high-performance PZT-5H/epoxy 1-3 composites, has a diameter of approximately 12.8 mm and a length of 11 mm. The method is simple to implement and suitable for the processing of radial transducers of any size. In addition, its performance, especially crosstalk, is simulated. According to the simulation results, array elements are precisely spaced at a pitch of 600 μm, which corresponds to one wavelength in water and is suitable for improving imaging capabilities. Given the requirements of narrow pitch and kerf width between adjacent elements, electrical connections were accomplished by a pre-designed flexible circuit with rectangular pads. The flexible circuit board is simple in design, cheap, and can realize precise excitation of piezoelectric elements. Lastly, the electrical and acoustic performance of the fabricated transducer, including impedance characteristics, pulse-echo response, and crosstalk, was characterized by adopting conventional test methods. The transducer mainly vibrates in thickness (*k*_33_) mode and exhibits a higher electromechanical coupling factor. Meanwhile, due to the larger kerf width, the crosstalk between array elements is also significantly reduced. These test outcomes demonstrate that the fabricated radial array transducer exhibits outstanding performance.

## 2. Materials and Fabrication Methods

For 1-3 composite consisting of ceramic and epoxy, piezoelectric ceramic square columns are actual sound-emitting and receiving components. Ideally, these columns should vibrate in the *k*_33_ mode. Then, the ceramic and epoxy 1-3 composite can achieve a larger electromechanical coupling factor *k_t_*. In this article, PZT-5H/epoxy 1-3 composite was chosen as the active material due to its superior electromechanical coupling coefficient. This characteristic signifies an excellent conversion efficiency between electrical and acoustic energies in a longitudinal resonance mode [9]. Moreover, the acoustic impedance of this composite is much closer to that of human tissues, making it ideal for broad bandwidth and high-sensitivity ultrasonic transducers. The composite utilized in our research was supplied by Zhongshan City Shengnuo Instrument Equipment Co., LTD (Zhongshan, China). The key property parameters of PZT-5H/epoxy 1-3 composite can be found in Table 1.

The characteristics of the PZT-5H composite-based material were instrumental in refining the design parameters for our circular array transducer. This optimization process encompasses the thicknesses of the piezoelectric layer, the backing layer, and the dimensions of each element [6], as referenced in Table 2. The simulation software employed for this task included PiezoCAD (version 4.01.), which is based on the Krimboltz, Leedom, and Mattaei (KLM) model (developed by Sonic Concepts, Woodinville, WA, USA), and the finite element analysis software Comsol6.2. Given the small acoustic impedance discrepancy between the PZT-5H/epoxy 1-3 composite and water, the matching layer is neglected. The final design parameters for the transducer are detailed in Table 2. The pitch of the radial array transducer was designed to be λ for the suppression of grating lobes during beam steering. Furthermore, to reduce crosstalk between adjacent elements, the kerf size was intentionally made larger.

**Figure 1:**
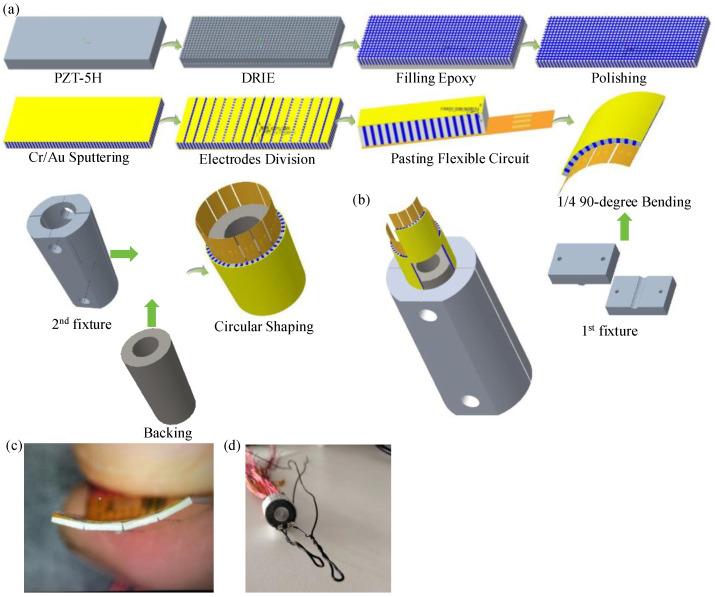
Fabrication of the radial array transducer: (**a**) Schematic of the configuration of developed 64-element circular transducer using PZT-5H 1-3 composite; (**b**) decomposition view of fabricating 64-element circular transducer using the 2nd fixture; (**c**) 1/4 90-degree transducer after bending; (**d**) prototype photo of the fabricated circular array transducer.

Figure 1 shows the fabrication procedure of the radial array transducer. Firstly, the 1-3 piezocomposite was fabricated based on a PZT-5H plate. In order to reduce the coupling effect of the transverse vibration mode, the ratio of height to width for the embedded ceramic rods should generally be as large as possible [10]. Considering the fragility of piezoelectric ceramics, the composite with the desired aspect ratio was prepared by the dice-and-fill method. The specific preparation process is also illustrated in Figure 1a. Specifically, the widths of piezoelectric columns and filled epoxy are, respectively, 120 μm and 80 μm. According to the central frequency parameter, the piezoelectric composite sample was prepared at the desired thickness of about 620 μm. The thickness of the radial array transducer has been approximately optimized to half the wavelength (λ/2), with λ representing the wavelength at the resonant frequency.

Both the top and bottom sides of the piezoelectric composite sample were then mechanically polished and sputtered with a Cr/Au (50/100 nm) electrode. The dimensions of the manufactured composite are 45 × 45 mm^2^. Subsequently, the composite was cut into multiple separate sections (1/4 planar unit) with a length of 9.6 mm and a width of 11 mm. After that, four 1/4 planar units were picked. The negative electrode of the 1/4 planar unit was cut into 16 elements. Then, the dispersed four 1/4 planar units were adhered to flexible circuit boards using insulating epoxy (EPO-TEK 301, Epoxy Technology, Billerica, MA, USA). Flexible circuits (10 × 15 mm^2^) were used to simplify the electrical connection to the separated elements. The length of the flexible circuit board, 10 mm, is approximately equal to that of a 1/4 planar unit in order to allow it to be inserted inside the capsules.

However, its width, 15 mm, is larger than that of a 1/4 planar unit in order to ensure a successful electric connection between all transducer elements and excitation signals. The width of each rectangular pad is 270 μm, which constitutes 68% of the width of the piezoelectric elements. In order to ensure optimal contact between the piezoelectric elements and pads, the length of the pads was set to 1 mm. Furthermore, all rectangular pads in flexible circuits should be aligned with the elements. An external stress was imposed on the 1-3 composite by a custom-made fixture in order to ensure thin bonding layers (3–5 μm). Concretely, each 1/4 planar unit attached with a flexible circuit board was clamped on two stainless cuboids assisted with fastening thread at 40 °C for 2 h. After 24 h, screw fastening nuts with a diameter of 3 mm were placed into the through-hole of the 1st fixture and tightened slightly at 80 °C. Then, the dispersed four parts can be bent to a 90-degree angle by exploiting the 1st fixture.

To shorten the ring downtime and reduce the reverberation of ultrasonic transducers, a backing layer was added. It should be noted that using the backing layer can increase the bandwidth of the transducer while sacrificing a part of the acoustic energy. Therefore, a moderate value of acoustic impedance for the backing layer (5.8 MRrayls) was selected to balance the bandwidth and sensitivity of the radial array transducer.

Using the EPO-TEK 301 and the 2nd fixture, four 90-degree curved modules were pasted onto the prepared backing layer and assembled into radial array transducers with 64 elements. Meanwhile, nuts with a diameter of 5 mm were also utilized to apply radial pressure in order to ensure that four 90-degree curved modules could be successfully attached to the backing layer. Additionally, there are four 90-degree alignment lines on the top surface of the 2nd fixture to guarantee the precise conjunction of four independent units. In order to easily observe the position of four 90-degree curved components during the experiment and adjust them accurately, the 2nd fixture was set to be transparent. Figure 1b illustrates the decomposition view of fabricating a 64-element circular transducer using the 2nd fixture. A metal pillar was placed inside the backing layer to support the transducer.

Figure 1c shows the 90-degree transducer after bending. As shown in Figure 1c, gaps exist in the 90-degree warped transducer. EPO-TEK 301 was used to fill in the spaces between different elements. The inner electrode of the radial array transducer was connected to electric excitation signals. The outer electrode was regarded as ground. In order to ensure that the outer electrodes of the 64 elements are interconnected, E-solder 3022 was applied. Finally, a 10 μm thick parylene (Parylene C, Specialty Coating Systems, Indianapolis, IN, USA) layer was vapor-deposited onto the external surface of the transducer by a parylene coater (Diener P6, Polyp-xylene coating system, Diener electronic, Ebhausen, Germany). The parylene acts as a matching layer between the designed radial array transducer and operating medium in order to coordinate their acoustic impedances. This parylene layer can also be used to protect wire bonds.

The fabricated radial array transducer, depicted in Figure 1d, features lead-out wires comprising 64 signal wires and 2 ground wires. Each array element, with a width of 400 μm, is composed of two rows of piezoelectric pillars and two rows of passive epoxy pillars. To minimize electric interference between adjacent elements, the kerf is designed to include one row of piezoelectric pillars and one row of passive epoxy pillars, with a width of 200 μm. Consequently, the center-to-center distance between two consecutive array elements is set at 600 μm, taking into account the fabrication challenges associated with electrical connections to small array elements and the stringent requirements of electrical systems. The spacing of electrical pads on the flexible circuit board is also maintained at 600 μm [6].

## 3. Characterization

This section describes how to calculate and measure key performance parameters of ultrasonic transducers. Firstly, the effective electromechanical coupling coefficient, denoted as *k_eff_*, plays a pivotal role in characterizing the conversion efficiency between electrical and mechanical energies in ultrasonic transducers. The calculation of *k_eff_* is as follows:

(1) $$k_{eff}=\sqrt{1-\frac{f_{r}^{2}}{f_{a}^{2}}}$$

where *f_r_* and *f_a_*, respectively, stand for the resonance and anti-resonance frequencies of the transducer. The higher value of *k_eff_* typically represents a larger efficiency of electroacoustic energy transfer and broader bandwidth. The resonance and anti-resonance frequencies are determined from the impedance and phase spectra of the radial array transducer, which are measured using an impedance analyzer (E4990A from Keysight Technologies Inc., Santa Rosa, CA, USA). In addition, the initial peak of element oscillations is identified as the resonance frequency, where the impedance reaches the minimum. With the frequency further increasing, the impedance increases to the maximum, which corresponds to the anti-resonance frequency.

The longitudinal resolution in ultrasonic imaging, which is the capacity to discern objects that are in close proximity along the path of the ultrasound beam, can be deduced from the characteristics of the echo signal in both the time domain and frequency spectrum. The transducer’s pulse-echo response was evaluated in a deionized water bath at ambient temperature, as referenced in [11]. Barrel-shaped quartz blocks with varying internal diameters were crafted to facilitate the selection of a suitable reflector body for the designed ultrasonic transducer. The transducer was placed in the center of the chosen quartz reflector with a distance at the near field/far field transition point. By connecting to an ultrasound transducer analyzer (ProCheck SC5, Broadsound Corporation, Shenzhen, China), the active element was excited individually by a 1 J electrical impulse with a 500 Hz repetition rate and 50 Ω output impedance. The actuation voltage amplitude was set at −75 V. The frequency bandwidth, ranging from DC to 55 MHz, defines the spectrum of the ultrasound beam emitted by the transducer. The echo response was captured by the receiving module of the ultrasound transducer analyzer. The frequency domain pulse-echo response can also be acquired. The bandwidth of the transducer was ascertained from the −6 dB points on the frequency spectrum. We denote the lower and upper −6 dB frequencies as *f*_1_ and *f*_2_, respectively, which correspond to the frequencies where the displacement amplitude is 50% (6 dB) of its peak value. Then, the center frequency is given by

(2) $$f_{c}=\frac{f_{1}+f_{2}}{2}$$

The −6 dB bandwidth can be described as

(3) $$BW=\frac{f_{2}-f_{1}}{f_{c}}\times 100\%$$

The mechanical quality factor, *Q_m_*, significantly impacts both the waveform emitted by the transducer and the response curve observed during signal reception. Typically, *Q_m_* can be calculated as

(4) $$Q_{m}=\frac{f_{r}}{f_{2}-f_{1}}$$

The two-way insertion loss (*IL*), also known as the relative pulse-echo sensitivity, is the ratio of transducer output power *P_o_* to input power *P_i_* delivered to the transducer from a driving source. If the output resistance *R_o_* is assumed to be equal to the input resistance *R_i_*, the *IL* can be simplified as the ratio of the echo voltage *V_o_* to the excitation voltage *V_i_* [2].

(5) $$IL=10\log\left(\frac{P_{o}}{P_{i}}\right)=10\log\left(\frac{V_{o}^{2}/R_{0}}{V_{i}^{2}/R_{i}}\right)=20\log\left(\frac{V_{o}}{V_{i}}\right)$$

The radial array transducer was actuated by a function generator (RIGOL DG4162), which produced a 20-cycle sinusoidal pulse with a peak amplitude of *V*_1_ at the central frequency *f_c_*. In response to this excitation signal, the transducer would receive an echo signal with an amplitude of *V_o_*, as detected by an oscilloscope (SDS5104X) with an input impedance of 1 MΩ. For reference, the amplitude of the driving signal *V_i_* was measured under a 50 Ω impedance condition, as noted in [12].

Crosstalk arises from the vibrational coupling effect between an activated transducer element and its neighboring elements. When a voltage signal is applied to stimulate a transducer element, ultrasonic vibrations are generated through the *d*_33_ piezoelectric effect. These mechanical vibrations can travel to adjacent elements, leading to unintended interactions. After receiving a pulse signal reflected by an interface, an electric signal can be generated by exploiting the inverse piezoelectric effect. Crosstalk can also distort the directivity characteristic of aperture in array transducers, which in turn degrades the quality of ultrasound images, as indicated in [13]. The degree of electrical and acoustical separation between elements is quantified by measuring the crosstalk level. To evaluate the interference between adjacent elements, a simulation is conducted in the time domain where a single element is activated, and the electric voltage of neighboring, non-intentionally stimulated elements is calculated. This process helps determine the extent to which adjacent elements contribute to mechanical crosstalk in ultrasonic transducers. In finite element simulation analysis, three cycles of sinusoidal wave signal whose amplitude is *v*_0_ are supplied to activate an element and then the voltage amplitude of adjacent elements (*v*_a_) can be calculated. Based on these calculations, the crosstalk level (CTL) is defined by the following equation [14,15]:

(6) $$\mathrm{CTL}=20\log\frac{v_{a}}{v_{0}}$$

## 4. Results and Discussion

### 4.1. Pulse Echo Simulation

**Figure 2:**
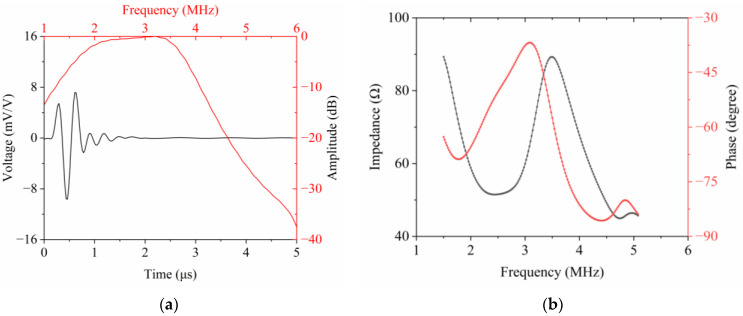
Simulated results obtained from PiezoCAD: (**a**) pulse-echo response; (**b**) electrical impedance magnitude and phase angle.

The pulse-echo waveform of the radial array transducer was simulated using the circuit-analogous model (PiezoCAD, sonic concepts, Woodinville, WA, USA) [16]. The time domain pulse-echo response and normalized frequency spectrum of a representative element in the radial array transducer are shown in Figure 2a using parameters in Table 1. It can be concluded that acceptable pulse and spectrum shapes were acquired. The center frequency and −6 dB BW are 2.69 MHz and 87.9%, respectively. The −6 dB BW is relatively high. The observed effect may be attributed to the larger dimension of the single transducer element along the elevation direction. Figure 2b illustrates the simulated frequency dependence of electrical impedance and phase of a single transducer element made of PZT-5H/epoxy 1-3 composite. It can be observed that the simulated resonance frequency and anti-resonance frequency are, respectively, 2.44 MHz and 3.50 MHz. According to Equation (1), *k_eff_* can be derived, which is 0.72. The simulated electrical impedance magnitude at the resonance frequency is 51.55 Ω which is close to 50 Ω. Accordingly, no additional electronic impedance-matching circuits between the transducer array and flexible circuit board are required to realize impedance matching. It is advantageous not only for streamlining the circuit design but also for enhancing the energy transfer efficiency between the radial array transducer and the electrical driving system.

### 4.2. Beam Profile

**Figure 3:**
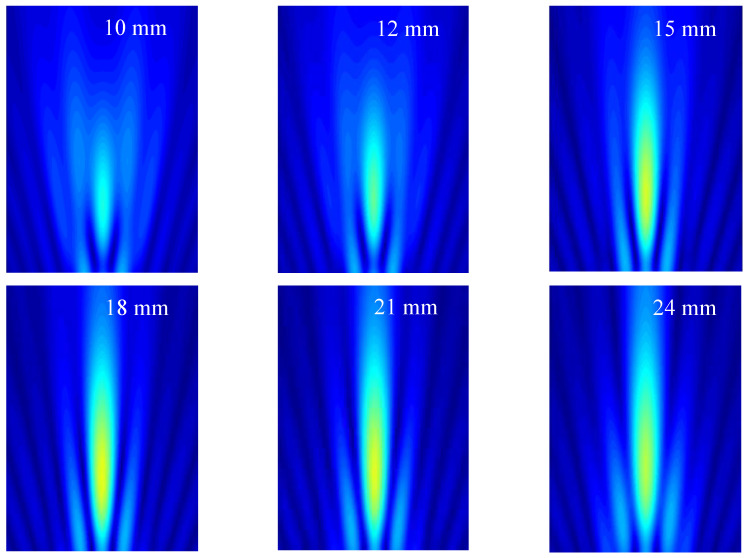
Acoustic field distribution for the designed radial array transducer with the focal points at 10 mm, 12 mm, 15 mm, 18 mm, 21 mm, and 24 mm above the *z*-axis.

Within the simulation model, the acoustic field characteristic of the radial array transducer can be clarified by sequentially applying delayed excitation signals to each of the 64 array elements. This controlled excitation strategy enables the focusing of the acoustic signal at a predetermined position. In order to simplify the calculation model, a 90-degree bent 1/4 transducer unit is employed, which is illustrated in Figure 1c. By assigning different excitation signals with calculated time delays to all elements, acoustic beams can be freely focused to specific depths, for example at 10, 12, 15, 18, 21, and 24 mm along the *z*-axis. Figure 3 shows the focused acoustic beam profiles of 16-element transducer arrays in the *x*-*z* plane at varying focal depths. From Figure 3, it is evident that the proposed transducer exhibits superior dynamic focusing capabilities, especially when the focus depth exceeds 15 mm. Moreover, increasing the focal depth further has a negligible impact on the beam profile. These simulation results serve as a valuable reference in assessing the echo characteristics of the transducer during testing.

**Figure 4:**
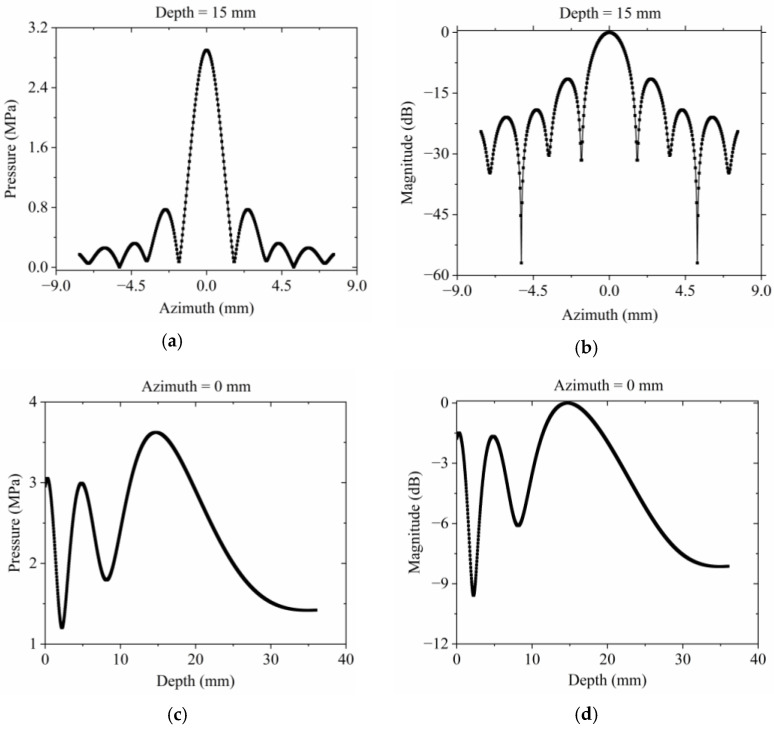
Distribution of acoustic pressure for the radial array transducer: (**a**) lateral sound pressure distribution at the focal depth 15 mm; (**b**) normalized lateral sound pressure distribution; (**c**) axial sound pressure distribution along the *z*-axis at *x* = 0 mm; (**d**) the corresponding normalized axial sound pressure distribution.

The detailed acoustic pressure distribution of the radial array transducer can be extracted from the simulated beam profile presented in Figure 3 [13,17]. With a focal depth of 15 mm, the acoustic pressure curves along the axial and lateral directions are both depicted in Figure 4. Specifically, Figure 4a illustrates the lateral sound pressure distribution at the focal depth, while Figure 4b displays its normalized counterpart. From the latter, the −6 dB beam width of the radial array transducer is determined to be 1.03 mm. The axial pressure distribution along the *z*-axis at *x* = 0 mm is shown in Figure 4c. The corresponding normalized pressure curve in Figure 4d reveals a −3 dB depth of focus of 11.37 mm for the radial array transducer.

### 4.3. Vibration Analysis

**Figure 5:**
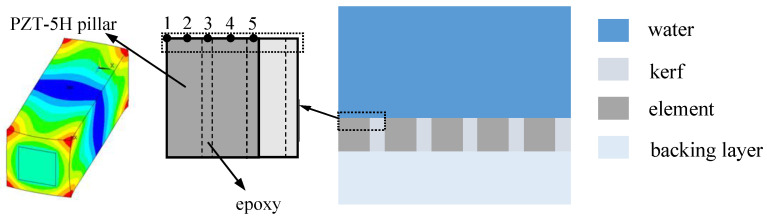
Simplified finite element model of the radial array transducer containing five elements.

Finite element simulation analysis was conducted to calculate the vibration properties of the designed transducer using the COMSOL6.2 software package. In order to exactly estimate the vibration performance of the radial array transducer, it is essential to carry out accurate structure modeling, meshing, boundary condition configuration, and solution processing [13]. Given that the radial array transducer comprises 64 elements, a simplified three-dimensional (3D) model was utilized for efficient electroacoustic and vibration simulations. Figure 5 displays a cross-sectional view of the 3D planar finite element model, which is based on the design parameters listed in Table 2. The simulation model includes five piezoelectric elements to facilitate further investigation into the crosstalk effect in subsequent studies. Moreover, in each piezoelectric element, there are two rows of piezoelectric pillars (PZT-5H) and two passive epoxy pillars. Using the simulation model depicted in Figure 5, the resonant frequency of the transducer’s thickness-stretch mode was determined. The calculated resonant frequency is found to be 2.52 MHz, which coincides with the results obtained in the Section 4.1. The distribution of spatial vibration displacement for a single piezoelectric element, calculated at the resonance frequency, is also depicted in Figure 5.

Theoretically, the PZT-5H square columns, being the primary vibration sources and considerably larger than the surrounding epoxy, should determine the resonance frequency of the radial array transducer. To confirm this hypothesis, a 3D finite element model was also created for a single piezoelectric pillar. The model yielded a calculated resonance frequency of 2.4 MHz, which aligns with the findings from the multiple-element model in Figure 5. Consequently, it is sufficient to evaluate the resonance frequency of a single piezoelectric element to gain a comprehensive understanding of the resonant characteristics of the entire array.

Understanding the vibration ways of individual elements in array transducers is crucial because of the vibration coupling phenomenon. Typically, the thickness extensional mode is the desired vibration mode for piezoelectric elements in array transducers. However, the generated longitudinal vibration energy can be transferred and dissipated between adjacent elements, potentially inducing undesired transverse vibrations. Theoretically, increasing the aspect ratio (height-to-width ratio) of piezoelectric ceramic rods embedded in composite materials can reduce the transverse vibration mode [18]. This study evaluates the vibration coupling characteristics of the array transducer by examining the response displacements of a piezoelectric element along the x and z axes. The upper surface of a single element was meshed, resulting in five computational nodes (from 1 to 5), as indicated by the dashed-line box in Figure 5. Because the kerf width is half of the element width, the kerf is meshed to generate two nodes (from 6 to 7). Five nodes were chosen to quantitatively assess displacement variations across different areas of the piezoelectric element. For the designed radial transducer, the thickness of piezoelectric square columns is 620 μm, which is nearly five times the width of 120 μm. Consequently, piezoelectric elements predominantly vibrate in the *k*_33_ mode [14], which enhances the electromechanical coupling factor of the 1-3 composite. In this paper, the parameter, *R*_disp_, is utilized to thoroughly assess the lateral effect of piezoelectric elements and is defined as

(7) $$R_{\mathrm{disp}}=\frac{Y_{\mathrm{disp}}}{X_{\mathrm{disp}}+Y_{\mathrm{disp}}}$$

In the above equation, *X*_disp_ denotes the transverse displacement of the designated nodes at center frequency. *Y*_disp_ corresponds to their longitudinal displacement. The *R*_disp_ serves as an indicator that reflects the purity of the longitudinal vibration mode and the concentration of longitudinal energy transfer. The calculated *R*_disp_ values for the chosen nodes are summarized in Table 3. According to the results in Table 3, it is evident that the predominant vibration mode for each node is *k*_33_. However, the presence of a parasitic length mode leads to a non-uniform distribution of node displacements along the horizontal direction.

**Figure 6:**
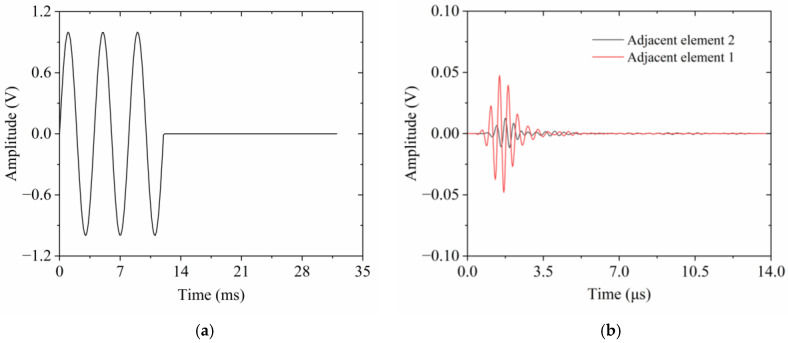
Crosstalk estimation: (**a**) initial excitation signal; (**b**) crosstalk signal of adjacent elements.

A pivotal parameter in characterizing the electrical properties of ultrasonic transducers that utilize 1-3 piezocomposites is crosstalk, stemming from the vibration coupling effect between the activated element and its neighbors. To quantify the interference level experienced by adjacent elements, a time-domain simulation is essential. This process involves the activation of a single element and the subsequent calculation of induced electrical voltage in the nonintentionally stimulated neighboring elements. In this research, crosstalk is also assessed through the finite element model depicted in Figure 5. A three-cycle sinusoidal wave voltage, as illustrated in Figure 6a, was employed to activate a representative element, which served as the reference signal. Assuming activation of the central element in the finite element model, the voltages generated in the two adjacent elements are extracted and presented in Figure 6b. Specifically, adjacent element 1 refers to the element closest to the excitation source. Adjacent element 2 stands for the element next closest to the activated element, corresponding to the leftmost and rightmost piezoelectric pillars shown in Figure 5. The maximum response voltages in these two adjacent elements are recorded as 0.047 V and 0.012 V, respectively. Substituting these values into Equation (6), the calculated CTLs for the adjacent elements are determined to be −26.56 dB and −38.42 dB, respectively.

### 4.4. Experimental Characterization

**Figure 7:**
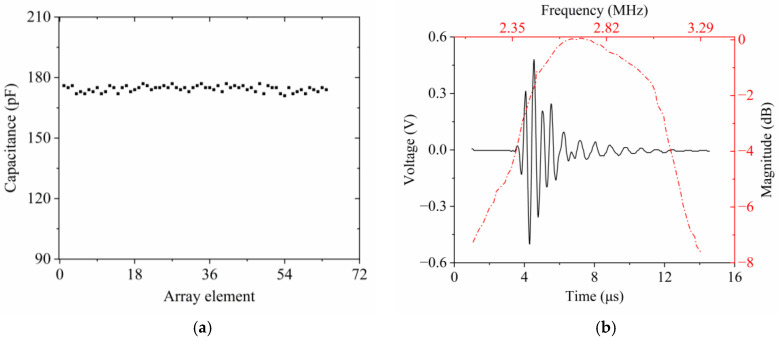
Tested results obtained from the ultrasound transducer analyzer ProCheck SC5: (**a**) element uniformity for capacitance; (**b**) pulse-echo waveform and frequency spectra of a single array element of the PZT-5H/epoxy 1-3 composite radial array transducer.

Figure 7a illustrates the uniformity of the capacitance across all elements, with an average capacitance of 178 pF. The tested pulse-echo response and its frequency spectrum of a typical piezoelectric element are shown in Figure 7b. The peak output voltage of the pulse-echo signal, measured at 0.51 V using the ProCheck SC5 (Broadsound Corporation, Jhubei, Taiwan)ultrasound transducer analyzer with a 1 μJ energy setting, signifies the transducer’s high sensitivity. Using Equation (2), the center frequency of the chosen element was found to be 2.70 MHz, which is very close to the resonance frequency. This experimental measurement is consistent with the resonant frequency predicted by finite element simulations. As observed in Figure 7b, the bandwidth can be derived from the echo response captured by the radial array transducer. Employing Equation (3), the average −6 dB bandwidth was determined to be 36%, which is acceptable for radial transducers. However, this bandwidth falls short of that achieved by rigid commercial probes which typically exhibit bandwidths exceeding 70% at lower frequencies. Notably, the measured bandwidth is indeed narrower than the values predicted by simulations. This discrepancy could be due to the metal support component embedded in the hollow backing layer, which may cause acoustic impedance mismatches, consequently leading to a reduction in bandwidth. Inconsistency in the epoxy coating, including EPO-TEK 301 and E-solder 3022, can also influence wave propagation, thereby potentially affecting the bandwidth. The omitted matching layer, crucial for impedance optimization, can significantly impact bandwidth characteristics. Furthermore, a simplified rectangular representation of the transducer element was established in PiezoCAD software. Future design iterations that incorporate more flexible matching layers specifically designed to adjust the acoustic impedance are anticipated to improve the bandwidth response. The average two-way insertion loss at the center frequency for the selected element was determined to be 31.86 dB, calculated using Equation (5).

**Figure 8:**
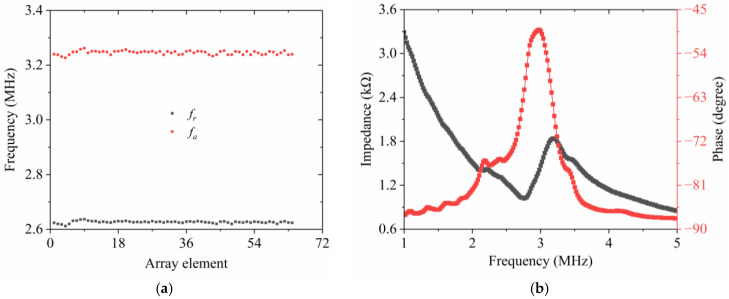
Measured results acquired from impedance analyzer: (**a**) element uniformity for resonance frequency and anti-resonance frequency; (**b**) electrical impedance and phase of a representative array element for the radial transducer.

Figure 8a depicts both the resonance and anti-resonance frequencies of all radial array elements. No open or shorted elements were detected. Then, the average resonance and anti-resonance frequencies of all transducer elements can be determined from these measurements using the following expression.

(8) $$\bar{f}=\sum_{i=1}^{64}f_{i}$$

As predicted by Equation (8), the average resonance frequency and anti-resonance frequency, measured across the 64 elements, are 2.71 MHz and 3.24 MHz, respectively. By substituting these calculated average resonance frequencies and anti-resonance frequencies into Equation (1), *k_eff_* can be deduced, which is found to be 0.55. These experimental results are in good agreement with the theoretical simulations. The computed *k_eff_* indicates a reasonably good electromechanical coupling capability for the fabricated ultrasonic transducer. The electrical impedance and phase response of a representative element are displayed in Figure 8b. At the resonance frequency, the electrical impedance of the measured element approaches 1 kΩ. The tested impedance value is relatively higher compared to the result obtained from simulations. This divergence is likely due to the small aperture size of the actual array element. Additionally, the presence of EPO-TEK 301 which has been filled within the transducer structure after bending also plays a role in influencing its impedance characteristics. Concurrently, to guarantee that the outer surface of the 64-element transducer shares a common ground, the unevenly coated conductive material, E-solder 3022, also alters the electrical impedance.

Table 4 provides a comparative analysis of critical performance parameters for the designed radial array transducer, highlighting a significant correlation between the experimental results and the simulation predictions. The relative error between the experimental and simulation values, *δ*, can be expressed as

(9) $$\delta =\left|\frac{T-S}{S}\right|\times 100\%$$

where *T* stands for the measured results, and *S* denotes the results acquired from the simulation software. As shown in Table 4, the calculated relative errors between the test results and the simulation results are small, except for the lower −6 dB frequency, *f*_1_. Actually, *f*_1_ was derived from the simulation software, PiezoCAD, as illustrated in Figure 2a. Therefore, the main reason for the large error in *f*_1_ is that the software cannot build an accurate radial array transducer model, but only the corresponding uncurved single plane transducer element. In addition, it may also be attributed to the larger dimension of the single transducer element along the elevation direction. The other observed discrepancies between the measured and simulated values may be attributed to the inhomogeneous flatness of the outer surface of the fabricated radial array transducer.

**Figure 9:**
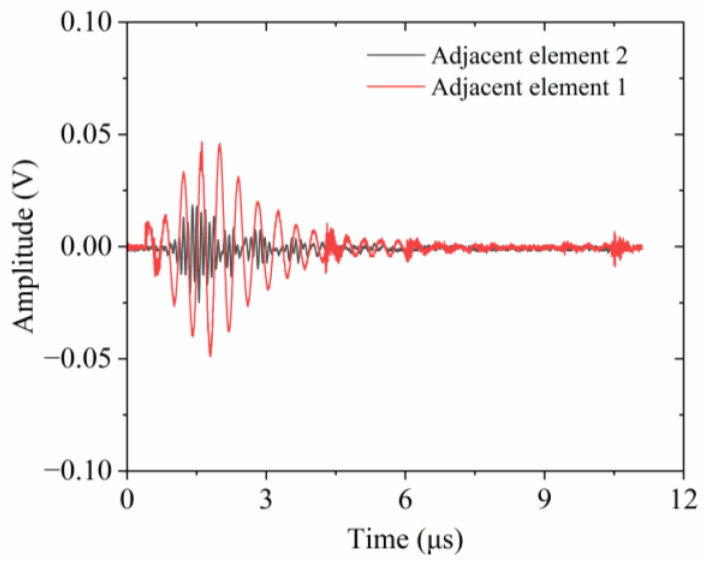
Crosstalk signals of adjacent elements.

Figure 9 describes the measured crosstalk signals across two neighboring elements. The peak voltage of the signal received by adjacent element 1 which is the closest to the active element is approximately 0.047 V. This is notably higher than the maximum voltage of 0.019 V detected by adjacent element 2, which is the next nearest element. The experimentally determined CTL for adjacent element 1 is −26.56 dB, which correlates well with the simulation data. In contrast, adjacent element 2 exhibits a substantially lower CTL of −34.42 dB. This reduction in CTL for adjacent element 2 indicates a significant attenuation in the interference caused by the activated element, suggesting that the coupling effect of transverse vibrations has been substantially mitigated.

## 5. Conclusions

A 2.5 MHz PZT-5H/epoxy 1-3 composite radial array transducer with 64 elements was designed and fabricated by a bending-and-superposition method. Array elements were spaced at a 600 μm pitch, equivalent to one wavelength in water. This configuration is instrumental in mitigating grating lobes for the presented radial transducers. The kerf width is 200 μm, which is beneficial in reducing the crosstalk level between array elements. A flexible circuit board with rectangular pads was designed and manufactured to simplify the electrical connection between the array elements and the driving system. Key performance parameters, including the pulse-echo response, impedance, acoustic field distribution, crosstalk, vibration mode, central frequency, etc., were all simulated to evaluate the behaviors of the radial array transducer. The simulated results suggest that piezoelectric elements in 1-3 composite mainly tend to vibrate in the *k*_33_ mode, and a larger electromechanical coupling factor *k_t_* can be achieved. The outer diameter of the manufactured transducer is around 12.8 mm. The fabricated transducer manifests a low two-way insertion loss of 31.86 dB. The −6 dB bandwidth is 36%, which is reasonable for radial transducers. At the resonance frequency of 2.71 MHz, the electrical impedance of the measured element is around 1 kΩ. Meanwhile, the decrease in CTL for adjacent element 2 signifies a marked reduction in interference from the activated element.

## Figures and Tables

**Table 1:** Properties of PZT-5H/epoxy 1-3 composite.

Specifications	Values
Density *ρ* (kg/m^3^)	3400
Electromechanical coupling coefficient *k*_t_	0.74
Acoustic impedance *Z*_c_ (MRayls)	13.43
Acoustic velocity *c* (m/s)	3950
Relative clamped permittivity	479
Thickness of piezoelectric layer (μm)	620
Volume ratio of piezoelectric ceramic	0.36

**Table 2:** Parameters of the circular array transducer.

Specifications	Values
Center frequency (MHz)	2.5
Element width (μm)	400
Pitch (μm)	600
Elevation dimension (mm)	11
Azimuth dimension (mm)	40
Thickness of backing layer (mm)	3
Number of elements	64

**Table 3:** Simulation results of *R*_disp_ for phased arrays.

Node	1	2	3	4	5	Average
*R*_disp_	0.90	0.86	0.81	0.78	0.75	0.82

**Table 4:** Comparison of main parameters acquired from simulation and experiment.

Results	*f_r_* (MHz)	*f_a_* (MHz)	*k_eff_*	*f*_1_ (MHz)	*f*_2_ (MHz)	*f_c_*
Simulation	2.44	3.50	0.72	1.40	3.92	2.66
Experiment	2.71	3.24	0.55	2.23	3.21	2.72
Relative error	11%	7%	24%	59%	18%	2%

## Data Availability

Data available on request from the authors.
